# Prevalence of Internet Gaming Disorder Among Intermediate and High School Students in Albaha, Saudi Arabia: A Cross-Sectional Study

**DOI:** 10.7759/cureus.37115

**Published:** 2023-04-04

**Authors:** Majed H Alghamdi, Majed M Alghamdi

**Affiliations:** 1 Preventive Medicine, Ministry of Health, Jeddah, SAU; 2 General Directorate of Health Affairs, Ministry of Health, Albaha, SAU

**Keywords:** gaming disorder, albaha, saudi arabia, excessive gaming, gaming addiction, internet gaming disorder

## Abstract

Background and Objectives

Excessive video game use, particularly among young people, is a growing problem that poses potential serious mental health risks in many parts of the world. However, there is a lack of research on the prevalence of internet gaming disorder (IGD) in Saudi Arabia, particularly in Albaha region. The objective of this study was to determine the prevalence of IGD among a sample of intermediate and high school students in Albaha and to identify potential factors associated with the development of the disorder.

Methods

In this cross-sectional study, we collected data between August and November 2022 using a self-administered online Arabic questionnaire, which included a validated translation of the IGD-20 test, which is a tool based on the DSM-5 criteria for diagnosing IGD. We used a multistage sampling technique, with two administrative areas as clusters, to randomly select eight intermediate and high schools with an equal distribution of male and female students. We analyzed the data using descriptive statistics and chi-square analysis.

Results

A total of 391 participants were included in the study, with an age range of 12 to 18 years. Males accounted for 51.4% (n=201) of the sample, and females accounted for 48.6% (n=190). The prevalence of IGD was found to be 3.5% (n=14), with males accounting for 64% (n=9) of affected participants. The study found that prolonged gaming duration (three or more hours per day), using mobile phones for gaming, and engaging in online gaming were significant factors associated with the diagnosis of IGD (p<0.001, p<0.001, and p=0.004, respectively).

Conclusion

This study provides preliminary evidence on the prevalence of IGD among intermediate and high school students in Albaha, Saudi Arabia. The results suggest a lower incidence of IGD compared to studies conducted in other regions of the country. A larger study with in-person interviews is needed to confirm the findings and extend the generalizability of the results. Furthermore, the study highlights the need for further research to explore the risk factors associated with IGD and to develop interventions to address this emerging mental health issue among Saudi Arabian youth.

## Introduction

The widespread use of video games as a form of entertainment among young people globally has become increasingly apparent. Numerous studies have indicated that a significant portion of children and adolescents spend substantial amounts of their awake hours interacting with electronic entertainment, including video games [1]. While video gaming is often considered a fun and stimulating leisure activity for most people, it can become a seriously addictive and even debilitating behavior for certain individuals [2,3].

The recognition of the potential impact of video gaming on mental health has led to the inclusion of internet gaming disorder (IGD) in the fifth edition of the Diagnostic and Statistical Manual of Mental Disorders (DSM-5) [3]. The DSM-5 defines IGD as “persistent and recurrent use of the Internet to engage in games, often with other players, leading to clinically significant impairment or distress.” Symptoms include preoccupation, withdrawal, tolerance, loss of control, loss of interest, continued use despite knowledge of psychosocial problems, deception, escapism, and jeopardizing or losing relationships or opportunities due to gaming [3,4].

The 11th Revision of the International Classification of Diseases (ICD-11) also recognizes the concept of gaming disorder [4]. Research suggests that IGD shares common features with other addictive disorders, such as substance abuse and gambling [5]. While DSM-5 diagnostic criteria do not differentiate between online and offline gaming, studies suggest that the criteria may be applicable to both [6]. Associations with IGD have been described in the literature and include gender, type of devices and games played, sociodemographic factors, level of education, and mood disorders such as depression and anxiety [5,7-10].

The estimated prevalence of IGD has varied greatly among studies conducted in different countries and specific populations, with some estimates ranging from less than 1% to more than 50% [11]. This variability in estimates may be due to differences in study design, population characteristics, cultural attitudes toward video gaming, and the diagnostic instruments used to assess IGD.

Limited research has been carried out in Saudi Arabia to investigate IGD, with the findings being insufficient and in need of further exploration [11-15]. Albaha region, one of the major administrative areas of Saudi Arabia, is located in the southeastern part of the country. With 35 intermediate and high schools, it caters to the education of more than 24,000 students [16]. To the best of our knowledge, there have been no prior studies conducted in the region to investigate the prevalence and associated issues of IGD. Therefore, the present study aimed to determine the prevalence of IGD among a sample of intermediate and high school students in Albaha region of Saudi Arabia and to identify potential factors associated with the development of the disorder.

## Materials and methods

This study used a cross-sectional design and was conducted in Albaha region. The data collection took place between August 2022 and November 2022 in eight randomly selected schools (four intermediate and four high schools, with an equal distribution of males and females) in Albaha region.

The data collection was conducted using a self-administered online Arabic questionnaire that consisted of three sections. The first section collected anonymous demographic information, such as age, gender, and grade level. The second section focused on gaming behaviors, including gaming platforms, frequency, duration, and devices used for gaming. The third section used a validated Arabic version of the IGD-20 test, which was developed by Pontes et al. in English to diagnose IGD based on six first-order latent domains that are typically present in behavioral addictions [17].

These six domains are salience, mood modification, tolerance, withdrawal symptoms, conflict, and relapse. Salience refers to the increasing importance of gaming in one's life, mood modification refers to the use of gaming to alter one's emotional state, tolerance refers to the need to spend increasing amounts of time gaming, withdrawal symptoms refer to the distress experienced when unable to play games, conflict refers to the negative impact gaming has on other aspects of one's life, and relapse refers to the return to gaming despite negative consequences. The IGD-20 test is widely used by other investigators; hence, this is why we chose to use it. The Arabic version of the IGD-20 test used in this study was translated and validated by Hawi and Samaha [18]. The participants rated all items of the test on a 5-point Likert scale, and a score of 71 or higher was considered diagnostic for IGD. A pilot study was conducted on 10 intermediate and high school students from the same area of the study to assess the clarity and appropriateness of the questions. The participants were asked to provide feedback on the wording and format of the questionnaire. Based on their feedback, minor modifications were made to the translation to adapt the tool for the target population.

We estimated the sample size for this study using the OpenEpi software [19], assuming a 50% prevalence of IGD in the absence of previous studies from Albaha, with a 95% confidence interval and a 10% non-response rate. The estimated sample size was 418 participants. The total number of intermediate and secondary school students in Albaha region is approximately 24,000 students, and we used the estimated prevalence rate to determine the appropriate sample size for the study.

A multistage sampling technique was used for this study. Albaha region has 35 intermediate and secondary schools that are divided into five administrative areas. Two administrative areas were randomly selected as clusters, and two intermediate schools (one for boys and one for girls) and two secondary schools (one for boys and one for girls) were randomly selected from each cluster. From each selected school, 52 students were randomly chosen while ensuring equal representation of all school levels. The online questionnaire was distributed to the selected schools, who then disseminated it to the eligible students.

The data collected from the online questionnaire was analyzed using IBM SPSS Statistics for Windows, Version 24.0 (IBM Corp., Armonk, NY). Descriptive statistics were used to analyze the data, including means, frequencies, standard deviations, and ranges. Chi-square tests were used to evaluate the associations between categorical variables, including comparing males with females in terms of gaming habits and identifying gaming habits and demographic associations with IGD diagnosis. The level of significance was set at p<0.05.

We obtained ethical approval (code A01314) from the Ministry of Health Institutional Review Board in Jeddah, and informed consent was obtained from both the participants and their parents or guardians. Only anonymous data were collected to ensure confidentiality. Administrative approval was granted by the Directorate of Education in Albaha, who provided us with a list of schools and the number of students at each level, which we used to design our study sample.

## Results

The study included 418 participants who were selected, of which 391 completed the questionnaire, yielding a response rate of 93.5%. Nearly half (48.6%) of the participants were female, and the average age was 15.6 years with a standard deviation of 1.7 years. The age range of the participants was 12 to 18 years. The details of participants' demographics are presented in Table 1.

**Table 1:** Demographic characteristics of study participants.

	Frequency (%)
Gender	
Male	201 (51.4%)
Female	190 (48.6%)
Grade level	
First year of intermediate school	59 (15.1%)
Second year of intermediate school	67 (17.1%)
Third year of intermediate school	64 (16.4%)
First year of high school	69 (17.6%)
Second year of high school	71 (18.2%)
Third year of high school	61 (15.6%)

The study found that 59% of the participants owned a gaming console, and 40% played games online. Additionally, the study found that 15% of participants played games online with friends, while 8% had friends that they only knew through online gaming and had not met in person. Around half of the participants did not play games daily, while the remaining participants reported playing games for varying durations. The details of participants' gaming habits are presented in Table 2.

**Table 2:** Gaming habits of study participants, stratified by gender and compared using chi-square tests.

	Male (n = 201)	Female (n = 190)	Total (n = 391)	P-value
Owns a dedicated gaming device/console	122 (61%)	107 (56%)	229 (59%)	0.379
Plays online games	94 (47%)	63 (33%)	157 (40%)	0.006
Uses phone for gaming	152 (76%)	93 (49%)	245 (63%)	<0.001
Plays online with friends	39 (19%)	19 (10%)	58 (15%)	0.009
Has a friend from online games	20 (10%)	12 (6%)	32 (8%)	0.190
Gaming screen time				
Does not play daily	95 (47%)	139 (73%)	234 (60%)	< .001
One hour or less	62 (31%)	36 (19%)	98 (25%)	
Two hours	14 (7%)	6 (3%)	20 (5%)	
Three hours	19 (9%)	5 (3%)	24 (6%)	
More than three hours	11 (5%)	4 (2%)	15 (4%)	

The study revealed an overall prevalence of 3.6% (95% confidence interval: 2% to 5.9%) for IGD in the sample, with 14 participants scoring 71 or higher on the IGD-20 test. Among these participants, five (36%) were female. All participants with IGD reported using their mobile phones for gaming along with at least one other device, and the most commonly used consoles were PlayStation 4 or 3. The majority (71%) of participants with IGD reported playing video games for more than three hours per day. Table 3 presents a summary of the findings related to participants diagnosed with IGD.

**Table 3:** Characteristics of participants who met the diagnostic criteria for internet gaming disorder.

	Male (n = 9)	Female (n = 5)	Total (n = 14)
Owns a gaming console	9 (100%)	5 (100%)	14 (100%)
Plays online games	8 (89%)	4 (80%)	12 (86%)
Uses phone for gaming	9 (100%)	5 (100%)	14 (100%)
Plays online with friends	6 (67%)	3 (60%)	9 (64%)
Has an online friend from gaming	5 (56%)	2 (40%)	7 (50%)
Gaming screen time			
Does not play daily	0 (0%)	0 (0%)	0 (0%)
One hour or less	0 (0%)	0 (0%)	0 (0%)
Two hours	1 (11%)	1 (20%)	2 (14%)
Three hours	1 (11%)	1 (20%)	2 (14%)
More than three hours	7 (78%)	3 (60%)	10 (71%)

The chi-square test was used to identify significant associations between IGD diagnosis and gaming-related variables as well as demographic variables. Participants diagnosed with IGD had a higher likelihood of playing for longer durations (p < 0.001), using their phones for gaming (p < 0.001), and engaging in online gaming (p = 0.004). However, gender, school level, and the type of device used for gaming did not show any significant association with the diagnosis of IGD in this study.

## Discussion

Our study on intermediate and high school students in Albaha region found a lower prevalence of IGD at 3.5% compared to other local studies conducted in Saudi Arabia. For instance, a study conducted in the Qassim region reported a 5% prevalence of gaming addiction among 2,675 intermediate and secondary school students using a 7-item scale [15]. Another study conducted in Faifa city using the same 20-item scale as our study reported a much higher prevalence of 29.3% [11]. Alsunni and Latif conducted a cross-sectional study on Saudi university students in 2022, and the reported prevalence of IGD was 21.5% in a sample of 843 students [13]. In another study involving 726 male high school students from Dammam city, the reported prevalence of IGD was 21.85% [12].

Various factors may have contributed to the variation in results. Our study's smaller sample size and specific demographics from Albaha region could have led to the lower prevalence rate of IGD. Differences in gaming culture and access to gaming devices and the internet across regions in Saudi Arabia could also account for variations. Differences in the methodology used to assess IGD could have influenced the findings as well.

The study identified prolonged gaming duration, using mobile phones for gaming, and participating in online gaming as significant factors associated with IGD. These findings are consistent with previous studies that have identified similar risk factors [5,7-10].

One limitation of the study is the relatively small sample size from two administrative areas of Albaha region, which may not be representative of the entire region. The study's reliance on self-reported measures may have led to reporting biases, and the cross-sectional design of the study limits causal inference. Another limitation is that the data collection was exclusively conducted online, potentially excluding students without internet access or those who preferred not to disclose their gaming habits online. Additionally, diagnosing IGD definitively requires assessment by a qualified psychiatrist. Finally, the study did not explore other potential risk factors for IGD, such as mood disorders and personality traits.

Future research should aim to increase sample size and geographic representation to better understand the prevalence of IGD in Albaha region. Longitudinal studies that track gaming behavior and its impact on academic and social outcomes could also provide valuable insights. Furthermore, it is important for future research to focus on further exploring the potential factors that may influence the prevalence of IGD. It may be worthwhile to investigate the potential impact of cultural and social factors, such as parental attitudes toward gaming and the availability of alternative leisure activities. Additionally, future studies could examine the long-term effects of IGD on academic performance, mental health, and social functioning.

Overall, this study provides a valuable contribution to the literature on IGD in Saudi Arabia and highlights the need for continued efforts to address this issue, particularly in Albaha region. By increasing awareness, promoting healthy gaming habits, and providing support for students who exhibit symptoms of IGD, we can help mitigate the potential negative consequences of excessive gaming.

## Conclusions

IGD is an emerging mental health concern with significant implications for all age groups, especially adolescents. Although our study found a relatively lower incidence of IGD among this population in Albaha region compared to other regions in Saudi Arabia, the limited sample size and use of only two out of the five administrative areas in the region as clusters restrict the generalizability of our findings. Further research is needed to better understand the prevalence and impact of this condition. Developing appropriate interventions and support systems, including promoting alternative leisure activities and educating on healthy gaming behaviors, is crucial in addressing this issue.
